# Maximal voluntary ventilation and forced vital capacity of pulmonary function are independent prognostic factors in colorectal cancer patients: A retrospective study of 2323 cases in a single-center of China

**DOI:** 10.1097/MD.0000000000025793

**Published:** 2021-05-21

**Authors:** Jiangpeng Wei, Ying Zhang, Pengfei Yu, Xiuqin li, Xiangying Feng, Shisen li, Gang Ji, Xiaohua Li

**Affiliations:** a Department of Radiotherapy, Xijing Hospital; b State Key Laboratory of Cancer Biology, National Clinical Research Center for Digestive Diseases and Xijing Hospital of Digestive Diseases, Fourth Military Medical University, Xi’an, Shaanxi; c The Air Force Hospital of Southern Theater Command, Guang zhou, Guangdong, China.

**Keywords:** colorectal cancer, maximal voluntary ventilation, pulmonary function, retrospective cohort, survival

## Abstract

Preoperative pulmonary function assessment is applied to select surgical candidates and predict the occurrence of postoperative complications. This present study enrolled 2323 colorectal cancer patients. Forced vital capacity (FVC) and maximal voluntary ventilation (MVV) were measured as predicted values. Associations between patient pulmonary function and both prognosis and postoperative complications was analyzed. The value of FVC and MVV optimal cutoff was 98.1 (*P* < .001) and 92.5 (*P* < .001), respectively. Low FVC and low MVV were associated with higher rates of postoperative fever (23.8% vs 13.9%, *P* < .001; 17.8% vs 13.3%, *P* = .049, respectively) and with higher rates of pneumonia (3.75% vs 1.73%, *P* = .002; 3.00% vs 1.71%, *P* = .009, respectively), pleural effusion (3.00% vs 1.57%, *P* = .033; 3.18% vs 1.42%, *P* = .006, respectively), and poor patient prognosis (5-year overall survival: 80.0% vs 90.3%, *P* < .001; 71.7% vs 91.9%, *P* < .001, respectively). In addition, low FVC was closely related to the higher rate of anastomosis leak (4.31% vs 2.29%, *P* = .013), low MVV was correlated with the higher rate of uroschesis (2.38% vs 0.65%, *P* < .001). In subgroup analyses, the predictive value of FVC and MVV in patients with different tumor stage was analyzed. Both low FVC and MVV were independent risk factors for poor prognosis in stage II and III, indicating that low FVC and MVV are predictive of poorer prognosis and higher risk of postoperative complications in colorectal cancer patients.

## 1 Introduction

Colorectal cancer (CRC) is the third most commonly diagnosed cancer and the fourth leading cause of cancer death in the world.^[1]^ Despite the rising incidence in several countries, CRC mortality rate is decreasing in many countries worldwide, due to the screening and improved surgical therapy.^[2,3]^ Surgeons commonly encounter patients suffering from impaired pulmonary function during preoperative evaluation. Postoperative pulmonary complications account for a substantial portion of the risks that are related to surgery and anesthesia and are a source of postoperative morbidity, mortality, and longer hospital stays.^[4]^ Therefore, pulmonary function testing is widely applied to select surgical candidates and predict the occurrence of postoperative respiratory complications,^[5]^ and it is even more likely than cardiac complications to predict long-term mortality after surgery.^[6]^ Some studies also revealed the influence of pulmonary function on abdominal surgery outcomes.^[7,8]^ It is difficult to diagnose pulmonary disease clinically, unless a patient presents overt respiratory symptoms. Therefore, preoperative screening for pulmonary disease usually depends on a given patient's previous medical history.^[9]^ In terms of evaluating pulmonary abnormalities and predicting postoperative complications, preoperative screening using pulmonary function testing is likely to be more valuable than conventional assessment.^[6]^ Although preoperative pulmonary function testing is accepted as an effective tool to predict operative risk before thoracic surgery,^[8]^ it is not yet routinely performed for colorectal cancer patients before surgery. However, the prognostic value of preoperative pulmonary function in CRC patients was often less investigated. The purpose of this retrospective study was to estimate the value of pulmonary function of CRC patients before surgery and to assess whether it has a prognostic value and the likelihood of postoperative complications.

## 2 Materials and methods

### 2.1 Patient selection

The retrospective study enrolled 2323 patients who were diagnosed with CRC and treated surgically between June, 2011 and December, 2016 at Division of Gastrointestinal Surgery, First Affiliated Hospital of Air Force Military Medical University. Enrolled patients were histologically confirmed and without distant metastasis. Patients with one of the following features, including (stage IV) CRC with more than one primary cancer, and with R1 or R2 resection, were excluded from our study. Other patients with missing data were also excluded. When the following data were available, patients were only considered eligible. According to the World Health Organization criteria, tumor differentiation grades were defined. Cancer staging was based on the American Joint Committee on Cancer Staging system (AJCC, 2002; Greene, American Joint Committee on Cancer, American Cancer Society, 2002). Pulmonary function test was carried out <7 days before surgery. FVC and MVV were measured by spirometry and as a percent of predicted values. The study was approved by the ethics committee of First Affiliated Hospital of Air Force Military Medical University. All patients provided written consent for storage of their information in the hospital database, and for the research use of the information.

### 2.2 Follow-up and outcome

Each patient was followed up periodically until death or April 2017 (every 3 months for the first 2 years, and every 6 months up to the fifth year) after surgery. The follow-up cycles varied from 3 to 6 months, with a median of 66.3 months. The follow-up visits consisted of a physical examination and laboratory studies at least every 6 months or when clinically indicated. The endpoint of the study was overall survival (OS). OS was calculated as the period from the date of diagnosis to the date of death from any cause or the date of last follow-up. Survival status was verified again by adopting the best available methods, including checking clinical attendance records and direct telecommunication with the patients or their families.

### 2.3 Statistical analysis

Chi-square test was conducted to compare categorical variables. Receiver operating characteristic curve (ROC) curve analysis was carried out to determine optimal cutoff values of FVC and MVV. If they have no homogeneity of variance, Kruskal Wallis test would be used. The survival rate was evaluated by Kaplane–Meier survival analysis, and their significance was calculated by the log-rank test. Univariate and multivariate Cox regression analyses were performed. Multivariable analyses were performed for factors that were significantly associated with OS in univariate analyses. All the statistical analyses were conducted using IBM SPSS 22.0 software. *P* value <.05 was considered to be statistically significant.

## 3 Results

**Figure 1:**
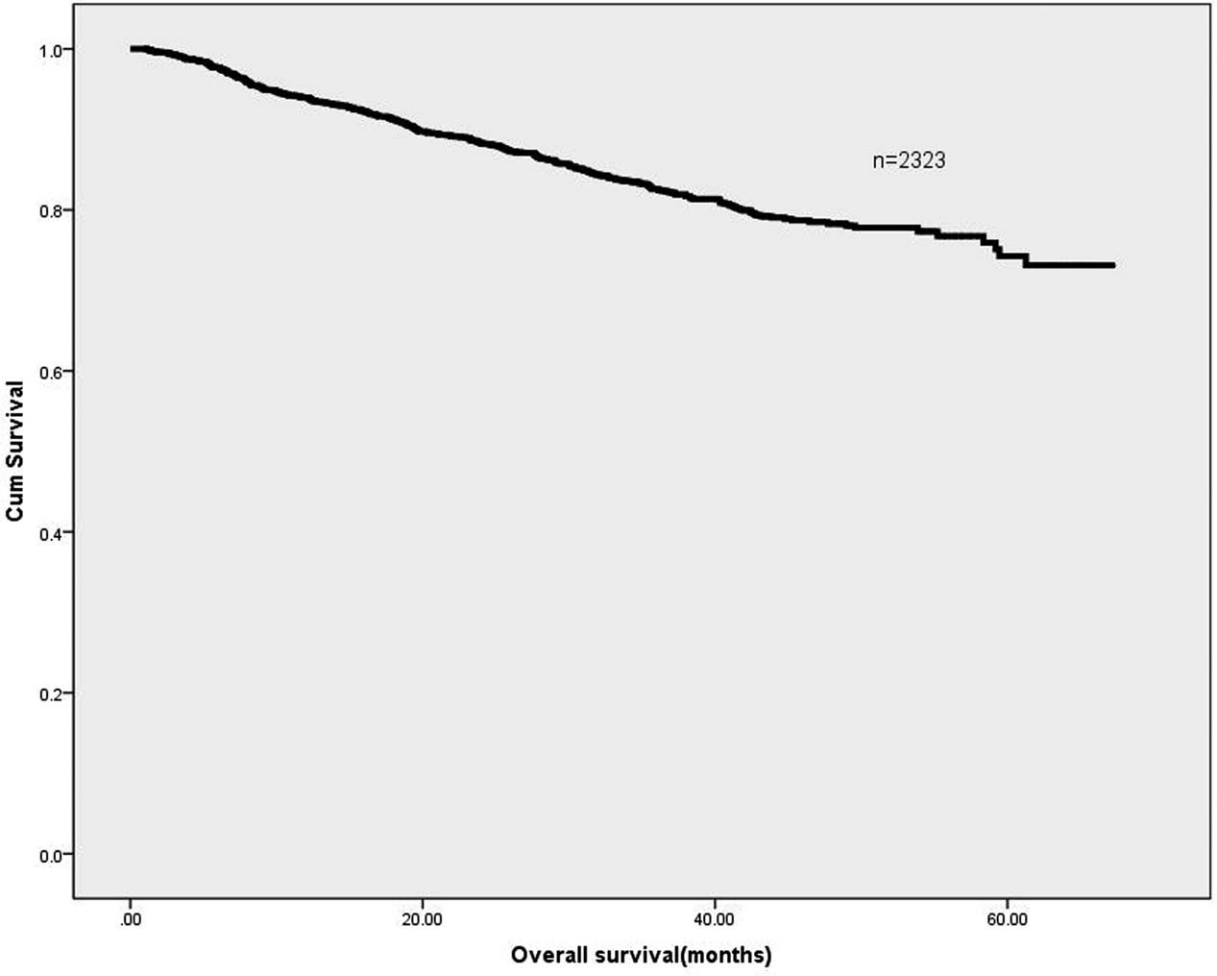
Overall survival of colorectal cancer patients.

Our study involved 1080 men (46.5%) and 1243 women (53.5%) CRC patients (Table 1). Median patient age was 59 years (range, 19–86), and median follow-up time was 28 months (range, 1–67). Patient 1-, 3-, and 5-year OS rates were 94.6%, 87.2%, and 85.5%, respectively (Fig. 1).

**Table 1:** Baseline characteristics of patients with low versus high FVC and MVV levels.

	FVC				MVV			
Patient characteristics	Low FVC	High FVC	*F* value	*P* value	Low MVV	High MVV	*F* value	*P* value
Gender			8.953	.003			66.993	<.001
Male	218	862			205	875		
Female	316	927			424	819		
Age, y			21.626	<.001			13.992	<.001
<60	238	1002			376	864		
≥60	296	787			253	828		
BMI			7.362	.025			9.444	.009
<18.5	84	236			101	219		
≥18.5–<25.0	328	1212			386	1154		
≥25.0	122	341			142	321		
Total protein			5.099	.024			16.141	<.001
<65.0	312	946			298	962		
≥65.0	222	843			331	734		
Albumin			7.257	.007			7.746	.005
<40.0	328	981			384	925		
≥40.0	206	808			245	769		
Tumor location^c^			79.353	<.001			54.734	<.001
Right hemicolon	142	419			128	433		
Left hemicolon	138	206			149	195		
Rectum (<6 cm)	108	496			150	454		
Rectum (≥6 cm)	146	668			202	612		
Tumor size, cm			6.743	.009			6.141	.013
≤5	256	972			359	869		
>5	278	817			270	825		
Pathology type			6.593	.037			33.387	<.001
Well differentiated	157	563			198	522		
Moderately differentiated	159	434			209	384		
Poorly differentiated	218	792			222	788		
pT status			9.491	.023			9.170	.027
pT1	47	238			82	203		
pT2	71	265			105	231		
pT3	181	563			213	531		
pT4	235	723			229	729		
pN status			5.522	.063			16.233	<.001
pN0	165	540			182	523		
pN1	221	662			209	674		
pN2	148	587			238	497		
pTNM stage			14.027	.001			14.965	.001
Stage I	113	526			210	429		
Stage II	190	565			189	566		
Stage III	231	698			230	699		

**Figure 2:**
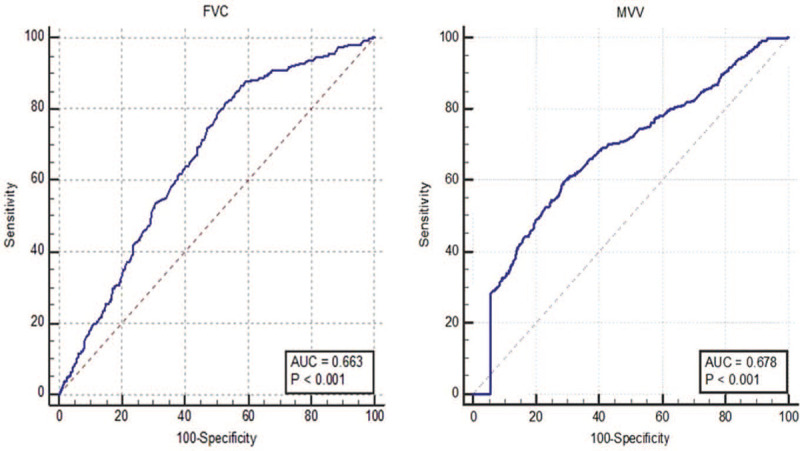
Optimal cutoff values of FVC and MVV were determined by ROC curve analysis. FVC = forced vital capacity, MVV = maximal voluntary ventilation, ROC = receiver operating characteristic curve.

FVC and MVV optimal cutoff values were 98.1 (*P* < .001) and 92.5 (*P* < .001) respectively (Fig. 2). Baseline characteristics of patients with low (FVC ≤ 98.1, MVV ≤ 92.5) versus high (FVC > 98.1, MVV > 92.5) FVC and MVV levels were analyzed and shown in Table 1. We discovered that lower FVC level was associated with female (*χ*^2^ = 8.953, *P* = .003), advanced age (*χ*^2^ = 21.626, *P* < .001), abnormal body-mass index (*χ*^2^ = 7.362, *P* = .025), abnormal total protein (*χ*^2^ = 5.099, *P* = .024), hypoproteinemia (*χ*^2^ = 7.257, *P* = .007), tumor location (*χ*^2^ = 79.353, *P* < .001), large tumor size (*χ*^2^ = 6.743, *P* = .009), advanced tumor depth (*χ*^2^ = 9.491, *P* = .023), poorly differentiated pathology type (*χ*^2^ = 6.593, *P* = .037) and advanced tumor stage (*χ*^2^ = 14.027, *P* = .001). lower MVV level was closely related to female (*χ*^2^ = 66.993, *P* < .001), advanced age (*χ*^2^ = 13.992, *P* < .001), abnormal body mass index (*χ*^2^ = 9.444, *P* = .009), abnormal total protein (*χ*^2^ = 16.141, *P* < .001), hypoproteinemia (*χ*^2^ = 7.746, *P* = .005), tumor location (*χ*^2^ = 54.734, *P* < .001), large tumor size (*χ*^2^ = 6.141, *P* = .013), advanced tumor depth (*χ*^2^ = 9.170, *P* = .027), poorly differentiated pathology type (*χ*^2^ = 33.387, *P* < .001), advanced lymph node metastasis (*χ*^2^ = 16.233, *P* < .001), and advanced tumor stage (*χ*^2^ = 14.965, *P* = .001) (Table 1).

**Figure 3:**
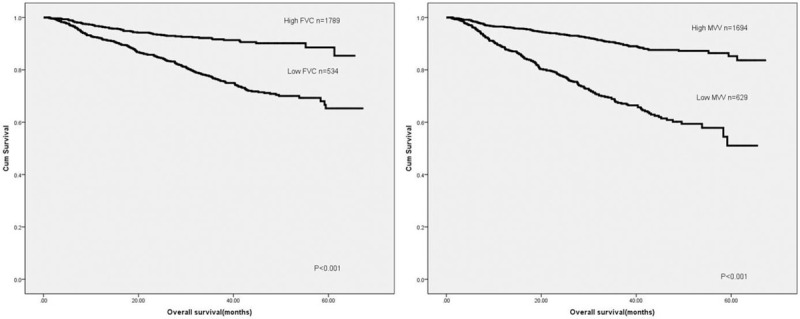
Patient overall survival based on FVC and MVV level. FVC = forced vital capacity, MVV = maximal voluntary ventilation.

**Figure 4:**
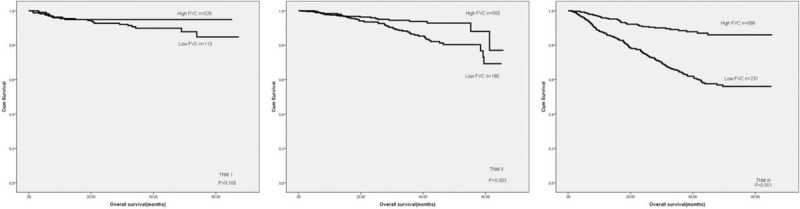
Overall survival of patients in different tumor stages based on FVC level. FVC = forced vital capacity.

**Figure 5:**
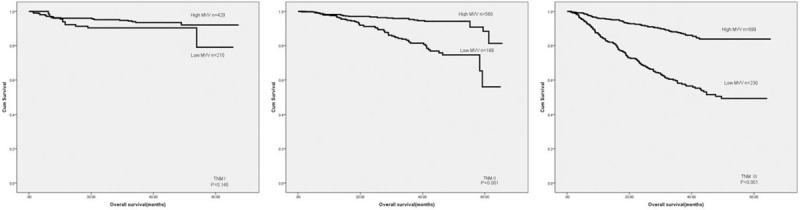
Overall survival of patients in different tumor stages based on MVV level. MVV = maximal voluntary ventilation.

Our results proved that low FVC (*P* < .001) and MVV (*P* < .001) were correlated with poor prognosis in CRC patients (Fig. 3). A univariate analysis demonstrated that tumor location, pathological type, tumor depth, tumor stage, FVC, MVV, total protein, and albumin were associated with prognosis (Table 2). Meanwhile, tumor depth, tumor stage, FVC, and albumin were independent prognostic predictors (Table 2). Subsequently, we then analyzed the predictive value of FVC and MVV in patients at different tumor stages. Both MVV and FVC were not related to prognosis in stage I CRC cases. However, low MVV and FVC were correlated with poor prognosis in patients at stage II and III colorectal cancer (Figs. 4 and 5). Univariate and multivariate analyses displayed that FVC and MVV was an independent risk factor for prognosis at stage II (Table 3) and III (Table 4) CRC patients.

**Table 2:** Univariate and multivariate Cox regression analysis for overall survival in patients with colorectal cancer.

	Univariate analysis			Multivariate analysis		
Characteristics	HR	95% CI	*P*^a^	HR	95% CI	*P*^a^
Gender			.427			—
Male	1.00	Reference		—	—	
Female	1.094	0.876–1.366		—	—	
Age (years)			.516			—
<60	1	Reference		—	—	
≥60	0.930	0.746–1.159		—	—	
Location			.043			.050
Right hemicolon	1	Reference		1	Reference	
Left hemicolon	0.683	0.499–0.935		0.695	0.513–0.943	
Rectum (≥6 cm)	0.922	0.668–1.272		0.928	0.673–1.278	
Rectum (<6 cm)	1.057	0.781–1.431		1.046	0.774–1.415	
Tumor size, cm			.601			
≤5	0.936	0.730–1.200				
>5	1	Reference				
Pathology (adenocarcinoma)			.018			.052
Well differentiated	0.657	0.442–0.979		0.648	0.437–0.962	
Moderately differentiated	0.690	0.528–0.901		0.683	0.530–0.898	
Poorly differentiated	1	Reference		1	Reference	
pT status			.001			<.001
pT1	0.244	0.089–0.668		0.318	0.142–0.713	
pT2	0.394	0.211–0.734		0.475	0.306–0.736	
pT3	0.641	0.487–0.842		0.618	0.472–0.808	
pT4	1	Reference		1	Reference	
pN status			.402			
pN0	1.00	Reference				
pN1	1.009	0.329–3.099				
pN2	0.667	0.268–1.663				
pTNM stage			.017			<.001
Stage I	0.538	0.218–1.330		0.390	0.292–0.520	
Stage II	0.695	0.532–0.908		0.695	0.533–0.905	
Stage III	1	Reference		1.00	Reference	
FVC			.020			.042
Low	1.00	Reference		1	Reference	
High	0.883	0.617–1.262		0.726	0.530–0.898	
MVV			.047			.046
Low	1.00	Reference		1.00	Reference	
High	0.795	0.634–0.997		0.896	0.834–0.980	
BMI			.380			
<18.5	1.105	0.566–0.901				
≥18.5–<25.0	0.951	0.817–1.062				
≥25.0	1	Reference				
Albumin			.007			.005
≥40.0	0.713	0.558–0.911		0.685	0.559–0.911	
<40.0	1	Reference		1	Reference	
Total protein			<.001			.231
≥65.0	0.528	0.390–0.714		0.825	0.794–0.920	
<65.0	1	Reference		1	Reference	

**Table 3:** Univariate and multivariate analysis of risk factors for prognosis of stage II colorectal cancer.

	Univariate analysis			Multivariate analysis		
Prognostic factors	β	HR (95% CI)	*P*	β	HR (95% CI)	*P*
Gender	0.312	1.366 (0.893–2.090)	.150			
Age	0.202	1.224 (0.813–1.842)	.004	0.244	1.276 (0.841–1.937)	.252
Location	–0.825	0.438 (0.270–0.710)	.001	–0.656	0.59 (0.274–0.984)	.0 45
Tumor size	0.343	1.409 (0.918–2.165)	.117			
Pathology	-0.163	0.849 (0.393–1.836)	.678			
pT status	-0.918	0.399 (0.241–0.662)	<.001	–1.331	0.264 (0.154–0.151)	.004
pN status	0.252	0.8281 (0.492–0.975)	.325			
FVC	0.788	2.198 (1.448–3.337)	<.001	0.765	2.149 (1.401–3.298)	.001
MVV	0.331	0.784 (0.478–0.780)	<.001	0.358	0.882 (0.517–1.367)	.017
BMI	–0.301	1.203 (0.764–1.625)	.784			
Albumin	0.082	0.957 (0.742–1.326)	.021	0.321	1.288 (0.825–1.873)	.032
Total protein	–0.038	0.824 (0.638–1.125)	.642			

**Table 4:** Univariate and multivariate analysis of risk factors for prognosis of stage III colorectal cancer.

	Univariate analysis			Multivariate analysis		
Prognostic factors	β	HR (95% CI)	*P*	β	HR (95% CI)	*P*
Gender	–0.038	0.963 (0.757–1.225)	.059			
Age	–0.208	0.972 (0.766–1.233)	.072			
Location	0.327	1.067 (0.764–1.490)	.007	–0.290	0.748 (0.521–1.076)	.040
Tumor size	–0.224	0.800 (0.620–1.031)	.005	–0.091	0.913 (0.696–1.197)	.509
Pathology	0.665	0.514 (0.331–0.799)	.003	–0.388	0.669 (0.425–1.052)	.007
pT status	–0.369	0.691 (0.531–0.900)	.006	–0.709	0.492 (0.271–0.894)	.020
pN status	–0.570	0.566 (0.445–0.719)	<.001	–0.471	0.624 (0.487–0.801)	.001
FVC	1.283	1.109 (0.768–1.704)	<.001	1.143	1.136 (0.897–1.896)	<.001
MVV	0.331	1.084 (0.878–1.280)	<.001	1.265	1.145 (0.755–1.778)	<.001
BMI	–0.545	1.124 (0.874–1.648)	.482			
Albumin	0.172	0.757 (0.442–1.026)	.042	–0.154	0.586 (0.373–0.894)	.082
Total protein	–0.385	0.784 (0.438–1.052)	.534			

Finally, we analyzed the relationships between FVC and MVV levels and postoperative complications (Table 5). Low FVC and low MVV indicate higher rates of pneumonia (3.75% vs 1.73%, *P* = .002; 3.00% vs 1.71%, *P* = .009, respectively), pleural effusion (3.00% vs 1.57%, *P* = .033; 3.18% vs 1.42%, *P* = .006, respectively). In addition, low FVC refers to a higher rate of anastomosis leak (4.31% vs 2.29%, *P* = .013) and low MVV represents a higher rate of uroschesis (2.38% vs 0.65%, *P* < .001).

**Table 5:** Comparison of postoperative complications.

	FVC				MVV			
Complications	≤98.1n = 534	>98.1n = 1789	*χ*^2^	*P*	≤92.5n = 629	>92.5n = 1694	*χ*^2^	*P*
Total cases	116	287	9.254	.002	138	265	12.681	<.001
Pneumonia	20	31	7.757	.005	22	29	6.812	.009
Pleural effusion	16	28	4.533	.033	20	24	7.671	.006
Wound infection	20	45	2.287	.130	13	52	1.696	.193
Fever	17	87	2.713	.100	28	76	0.001	.971
Anastomosis leak	23	41	6.234	.013	20	44	0.580	.446
Anastomosis bleeding	4	14	0.006	.938	6	12	0.360	.549
Chyle leakage	3	10	0.001	.994	6	7	2.409	.121
Ileus	6	12	1.096	.295	8	10	2.771	.096
Uroschesis	7	19	0.230	.632	15	11	12.482	<.001

## 4 Discussion

The association among preoperative pulmonary function and postoperative pulmonary complications and patient mortality have been well investigated. Feng et al^[9]^ reported that pulmonary disease was associated with postoperative morbidity in a large, multicenter, laparoscopic gastrectomy study. Jeong et al^[7]^ revealed that preoperative pulmonary function testing effectively predicted the risk of surgical complications and systemic complications in patients undergoing gastrectomy. The prognostic value of preoperative pulmonary function has mainly been investigated in thoracic surgery.^[7]^ Guo et al^[11]^ proved that FVC was an independent risk factor for the prognosis of non-small cell lung cancer patients who experienced curative resection, and FVC <80% predicted poor patient survival. Matsuzaki et al^[10]^ discovered that low forced expiratory volume 1 (FEV1)/FVC ratios with reduced overall and disease-free survival in lung cancer patients undergoing thoracic surgery. The same group revealed that the carbon monoxide diffusing capacity of the lung and the inspiratory capacity/total lung capacity ratio were associated with patient prognosis.^[10]^ However, the prognostic value of preoperative pulmonary function in colorectal cancer patients was rarely investigated. Sagawa et al^[13]^ illustrated that pulmonary dysfunction function is a risk factor for remote infections following surgery for CRC. Our study demonstrated that low FVC and MVV are related to poor prognosis in CRC patients, and both of them were independent prognostic predictors.

FVC is the ratio of actual vital capacity to predict vital capacity, and a FVC <80% is associated with restraint disorder.^[13]^ FVC reflects an individual's potential abilities such as exercise capacity,^[13]^ and values lower than 80% indicate the presence of restrictive impairment.^[13]^ The decreases in FVC postoperatively in patients who have preoperative restrictive impairment suggest that they may have developed a severe pulmonary complication. Particularly, atelectasis occurs due to suppressed deep breathing from pain. Tajima et al^[14]^ displayed that FVC may be a predictor of postoperative complications, especially pneumonia in colorectal cancer surgery. Multiple groups investigated the association between FVC and survival in the general population.^[15,16]^ Burnery et al^[16]^ reported that FVC, but not airway obstruction, can predict survival in asymptomatic adults without chronic respiratory diagnoses or persistent respiratory symptoms. Low FVC was closely related to increased mortality risk.^[17]^ We proposed 2 possible explanations for these findings, both of which strengthened the case for using pulmonary function testing in gastric cancer patients prior to surgery. First, pulmonary function tests may reflect muscle strength and general energy levels, and physical and psychological disorders may manifest as lower values. Therefore, these tests may indicate an individual patient's overall health.^[17]^ In these cases, FVC may reflect overall cardiopulmonary function as well as general health.

MVV is a pulmonary function test, and it can measure the maximum amount of air a person can inhale and then exhale with voluntary effort.^[20]^ In addition to the well-recognized clinicopathological prognostic factors, recent studies focused on identifying new factors, which can be highly reproducible, easily obtainable, inexpensive and reliable. We provided two possible explanations for these findings, both of which strengthened the case for using pulmonary function testing in gastric cancer patients prior to surgery. First, pulmonary function tests may reflect muscle strength and general energy levels, and physical and psychological disorders may manifest as lower values. Thus, these tests may indicate an individual patient's overall health. Second, poor fetal growth rates and lower birth weights may result in reduced lung function and increased risk of cardiovascular disease.^[18,19]^ In these cases, MVV may reflect overall cardiopulmonary function and general health as well.

Given the high operative mortality rate after surgery for CRC among patients who develop infectious complications. It was found that low FVC is related to pneumonia, pleural effusion and anastomosis leak, which may lead to severe abdominal infection. Preoperative pulmonary function is included in the risk score of the estimation of physiologic ability and surgical stress scoring system for the assessment of surgical risk proposed by Mcmillan^[21]^ with the reference values of FVC <60% and/or FEV1.0% <50%. Although these values are stricter than those applied in the present study, pulmonary function was a risk factor for postoperative complications. We hypothesized that pulmonary dysfunction can cause pulmonary complications and an oxygen supply disorder, and circulatory disorders might make it difficult to supply sufficient energy for tissue repair. As a result, patients suffering from pulmonary dysfunction might develop anastomosis leak at a high rate.

There were several limitations in our present study. Firstly, it was a retrospective analysis and limited to a single center. Multicenter studies are needed to verify the predictive value of MVV. Secondly, our patient cohort was not large enough, and small sample sizes can result in biased statistical analyses. Thirdly, postoperative pulmonary function may play roles in gastric cancer patient prognosis, and should be explored.

In conclusion, our study demonstrated that low FVC and MVV were closely related to poor prognosis in colorectal cancer patients, and FVC and MVV were independent prognostic predictors.

## Author contributions

**Data curation:** Jiangpeng Wei, Ying Zhang, Pengfei Yu.

**Formal analysis:** Jiangpeng Wei, Pengfei Yu, Shisen li, Xiaohua Li.

**Funding acquisition:** Xiangying Feng, Xiaohua Li.

**Investigation:** Jiangpeng Wei, Xiuqin Li.

**Methodology:** Xiuqin Li.

**Project administration:** Jiangpeng Wei, Shisen li, Gang Ji.

**Resources:** Jiangpeng Wei, Ying Zhang, Xiuqin Li, Xiangying Feng, Gang Ji.

**Software:** Jiangpeng Wei, Pengfei Yu, Xiangying Feng, Xiaohua Li.

**Supervision:** Jiangpeng Wei, Xiaohua Li.

**Visualization:** Xiuqin Li, Shisen li, Xiaohua Li.

**Writing – original draft:** Jiangpeng Wei, Pengfei Yu, Shisen li, Xiaohua Li.

**Writing – review & editing:** Jiangpeng Wei, Ying Zhang, Xiuqin Li, Gang Ji, Xiaohua Li.
